# Comment on ‘Molecular evidence of viral DNA in non-small cell lung cancer and non-neoplastic lung’

**DOI:** 10.1038/bjc.2016.338

**Published:** 2016-10-20

**Authors:** Antonio Ponzetto, Natale Figura, John Holton

**Affiliations:** 1 Department of Medical Sciences, University of Torino, Corso AM Dogliotti 14, Torino 10126, Italy; 2 Department of Medical, Surgical, Neurological Science, University Siena, viale Bracci 16, Siena 53100, Italy; 3 Mycobacterial Reference Unit, National Mycobacterium Reference Laboratory (NMRL), Abernethy Building, Institute of Cell and Molecular Science (ICMS), 2 Newark Street, London E1 2AT, UK

**Sir,**

The very interesting paper by Robinson *et al* (2016) is particularly intriguing for gastroenterologists. We have often encountered cases of non-small cell lung cancer (NSCLC) in patients that we have treated for Hepatitis B Virus (HBV)-related hepatocellular carcinoma (HCC). Regarding HCC, patient survival has been extended to 10 years, thanks to direct-acting antiviral drugs that suppress HBV replication, the ease of ablation of tumoural areas by radiofrequency, and the infrequent occurrence of metastasis. Conversely, the survival of NSCLC patients remains dismal. The co-occurrence of HCC with NSCLC has been dismissed as casual owing to the high prevalence of NSCLC in the heavily industrialised car-industry town of Turin and the high prevalence of HBV in Italy. The astonishing finding by Robinson *et al* (2016) of HBV sequences in 90% of squamous NSCLC is enlightening; indeed, it suggests a mechanism for preventing the squamous subtype of NSCLC by inhibiting HBV and any accompanying inflammatory mechanisms. Chronic HBV infection alone is a slow carcinogen, but its efficacy is increased by pro-inflammatory mechanisms, one of them being the bacterium *Helicobacter pylori* (Hp); Hp has been shown to have a role in causing both HCC (Ward *et al*, 1994) and lung cancer (Deng *et al*, 2013), and the reported association of Hp infection with the risk of developing lung cancer is 5–10 times stronger than with passive smoking exposure (Deng *et al*, 2013). The cumulative carcinogenic effects of more than one infectious pathogen could be more than additive; this could explain the occurrence of NSCLC in never smokers. HBV and Hp are widespread throughout the world and are known carcinogens; thus co-infection with both pathogens could amplify the chance of developing cancer. Hp causes gastric cancer and has also been found in the vast majority of patients with hepatitis virus-related HCC in Europe by us (Leone *et al*, 2003) and others, and particularly in China (Wang *et al*, 2013), adding to the notion that co-occurrence of these two pathogens tends to enhance neoplastic progression. Chronic inflammation represents a strong promoter of cancer (Balkwill and Mantovani, 2001), and pathogenic strains of Hp generate a pro-inflammatory status (Keates *et al*, 1997) capable of sustaining tumour amplification, acting at a distance. Indeed, a high inflammation score predicts the worst survival in all solid cancer types (Proctor *et al*, 2011). The cytotoxin-associated protein A (CagA) of Hp is transported throughout the body via microvesicles (Shimoda *et al*, 2016) and is a paradigm of hit-and-run carcinogenesis (Hatakeyama, 2014); in addition, CagA depletes the TP53 tumour-suppressor protein (Wei *et al*, 2015; Yong *et al*, 2015), which is missing or mutated in most lung and other cancers. In summary, both HBV and Hp infections can now be cured, if identified; hence we hypothesise that the low-cost screening for these two pathogens could reduce the huge suffering, medical costs, and staggering death toll owing to squamous NSCLCs.
